# Effective Treatment of Veterans With PTSD: Comparison Between Intensive Daily and Weekly EMDR Approaches

**DOI:** 10.3389/fpsyg.2018.01458

**Published:** 2018-08-24

**Authors:** E. C. Hurley

**Affiliations:** Soldier Center, Clarksville, TN, United States

**Keywords:** EMDR, PTSD, daily-treatment, moral injury, phantom limb pain, complex-PTSD

## Abstract

The effectiveness of EMDR therapy in treating veterans diagnosed with PTSD was evaluated in this study using two treatment formats: intensive daily EMDR treatment provided twice a day during a 10-day period and a second format of one session each week. The study used archived outcome data previously collected and stored at Soldier Center. Both formats provided 18–20 treatment sessions of EMDR therapy to veterans diagnosed with PTSD that included dissociative exhibitions and moral injury issues. Questions addressed included: (1) does EMDR therapy administered twice daily ameliorate veterans’ PTSD symptoms; (2) does EMDR therapy administered twice daily provide equivalent outcome results as EMDR therapy administered weekly for 18–20 sessions; and (3) does the treatment outcome persist. The effectiveness of the weekly treatment group was also evaluated. Both groups’ results were assessed at pre-treatment, post-treatment and 1-year follow-up. The results indicated that both weekly treatment and intensive daily treatment groups produced statistically significant treatment effects (*p* < 0.001) that were maintained at 1-year follow-up. The10-day EMDR intensive daily treatment (EMDR therapy twice a day for 10 days) produced a similar outcome as to that of the weekly treatment with a 1-year follow-up. Results support the effectiveness of EMDR therapy when offered in both weekly treatment format as well as the intensive 10-day format on an outpatient basis. While recognizing the limitations of this study the results are significant to warrant additional research.

## Introduction

With the United States and its allies currently fighting the longest war in its history, a record number of veterans with PTSD diagnoses continues to be reported by the Department of Veterans Affairs (Department of Veterans Affairs and Department of Defense, 2012). The toll of military service is documented as an average of 20 veterans from the U.S. military commit suicide each day (Department of Veterans Affairs [DVA], 2016a,b). Resources for attending to the mental health needs of veterans have been challenged for over a decade. A recent review of randomized controlled trials (RCT) in the treatment of military- related PTSD (Steenkamp et al., 2015), found the most widely disseminated trauma-focused interventions result in low remission rates. Specifically, “approximately two- thirds of patients receiving CPT (cognitive processing therapy) or prolonged exposure retained their PTSD diagnosis after treatment (range, 60–72%)” (p. 489). Foa et al. (2018) reported their findings in evaluating the effect of Prolonged Exposure and Present-Centered Therapy on the reduction of PTSD symptoms to be “severely modest” (p. 354). In addition, a mean 25% drop-out rate was reported and high dropout rates severely impact the mental health needs of veterans (Imel et al., 2013). Therefore, the testing of other evidence- based approaches, such as EMDR therapy, has been recommended (Steenkamp et al., 2015; Yehuda and Hoge, 2016; Kudler, 2017; Hoge and Chard, 2018).

Three decades of research have resulted in 30 randomized controlled trials (RCT) attesting to the efficacy of EMDR therapy in the treatment of PTSD. The Practice Guidelines of the World Health Organization [WHO] (2013) describe the EMDR therapy approach, “Like CBT with a trauma focus, EMDR aims to reduce subjective distress and strengthen adaptive beliefs related to the traumatic event. Unlike CBT with a trauma focus, EMDR does not involve (a) detailed descriptions of the event, (b) direct challenging of beliefs, (c) extended exposure, or (d) homework” (p. 1). Thirty RCTs have documented the positive effects of the bilateral stimulation used in the EMDR therapy (Lee and Cuijpers, 2013) and a meta-analysis (Swift and Greenberg, 2014) reported a high treatment retention rate. International professional organizations, including the American Psychiatric Association (American Psychiatric Association [APA], 2004); Department of Veterans Affairs and Department of Defense (2004, 2017); Institut national de la santé et de la recherche médicale (INSERM Collective Expertise Centre, 2000/2004); World Health Organization (World Health Organization [WHO], 2013) designate EMDR therapy as an effective trauma treatment. Yet, in spite of broad recognition of the effectiveness of EMDR therapy, it has been the subject of few veteran-focused research studies. In fact, excluding component analyses (e.g., Boudewyns and Hyer, 1996) and studies utilizing only two sessions (e.g., Jensen, 1994; Devilly et al., 1998), the only RCT evaluating EMDR therapy in the treatment of American veterans with PTSD was conducted in 1998 (Carlson et al., 1998). The treatment resulted in a 76% elimination of PTSD in 12 sessions, with a zero dropout rate. Comparable results have been reported from a variety of case series (Lipke and Botkin, 1992; Silver et al., 1995, 2008; Young, 1995; Howard and Cox, 2006; Wesson and Gould, 2009; Wright and Russell, 2012). In addition, EMDR has proven successful in the treatment of co-occurring phantom limb pain in military personnel (e.g., Russell, 2008).

While there has been limited evaluation of EMDR therapy in the treatment of veterans, since 2003 Soldier Center, a community-based outpatient treatment facility, has provided EMDR therapy to veterans. Treatment was initially offered to all clients at Soldier Center on a weekly basis. In 2005, the first intensive treatment, providing EMDR therapy twice a day for 5–10 days, was offered to veterans. This program evaluation study provides a retrospective evaluation of the data regarding both weekly and daily treatment regimes.

## Materials and Methods

Research data used in this program evaluation study was regularly collected and archived at Soldier Center as a means of evaluating treatment efficacy. Pre- and post-treatment measures with follow-up were routinely collected from all clients and evaluated to insure the effective treatment of clients. Participants were informed of the nature and purpose of gathering treatment outcome measures noting the use of the collected data would assist in providing effective of other veterans in the future. The importance of the veterans’ consent was stressed as permission to use the collected data in published research studies was verbally requested and received from the veterans. Participants were assured their treatment would not be influenced by whether or not they participated in data collection. The risk associated with treatment were provided as part of the informed consent form. Veterans were assured that their confidentiality would be maintained throughout the archival and evaluation process.

Data on 30 veterans’ treatment was collected and divided into two groups of 15 participants. one group was treated with EMDR therapy daily, twice a day over a period of 10 days. The other group was composed of data from veterans treated weekly. Both groups were treated a total of 18–20 sessions. The weekly therapy group consisted of veterans previously treated at veteran centers and local soldiers referred by the military Tricare office for their PTSD symptomatology. Once the data was retrieved, it included in an Excel PTSD spreadsheet containing the names, age, treatment dates, number of sessions and data from the PTSD Checklist (PCL-M/PCL-5), Impact of Events Scale-Revised (IES-R), and the Dissociative Experiences Scale-II (DES-II). The research questions were formulated as: (1) does EMDR therapy administered twice daily ameliorate veterans’ PTSD symptoms; (2) does EMDR therapy administered twice daily provide equivalent outcome results as EMDR therapy administered weekly for 18–20 sessions; and (3) does the treatment outcome persist.

Selection criteria limited participants to persons meeting diagnostic criteria for PTSD with no co-occurring disorders such as active addictions, chronic depression or suicidal/ homicidal intent. Persons were stable enough to self-regulate during the early history-taking and preparation phases of therapy. The first clients in the intensive daily group were active duty soldiers referred from Army Warrior Transition Units (WTU)s for an intensive 5-day treatment period. All participants demonstrated symptoms of complex PTSD.

According to state legislation and published ethical guidelines, an ethics committee does not need to review the data and approve an exemption for obtaining written informed consent in the use the archival data used in this study. Participants were informed that their treatment would not be influenced by either participation or non-participation in data collection. Accumulated data were gathered based on the clients’ willing participation in providing three-point (pre-, post-, and follow-up) data collection. Verbal information was provided regarding how the data would be used in the future to assist Soldier Center in evaluating and improving effective treatment to veterans. The treatment clients received was considered standard care. Fidelity checks, insuring standardized treatment, was conducted on all EMDR therapists by each therapist presenting videos of their psychotherapy sessions to the executive director. The same therapists provided treatment to both the intensive daily treatment and the weekly treatment groups. All active duty soldiers were authorized for PTSD treatment by Tricare. Data were collected by each providing therapist.

A description of the clinical protocol in this study is included under the **Supplementary Material** section. All participants completed a three-point assessment with pretreatment and post-treatment evaluations completed during their scheduled sessions. Response at follow-up differed with the two treatment groups. The 1-year follow-up evaluation return rate from members of the intensive daily group increased from an initial 28% when the entire packet (listed under measures) was sent and increased to 62% when only the IES-R was sent. In comparison, there was an initial response rate of 36% among the weekly participants at 1- year follow-up. The response increased to 58% when the completed IES-R measure was requested at 1-year follow-up. Dates for the intensive daily group covered the period of 2008–2016. Dates of treatment for the weekly treatment group occurred during an 18 month period, June 2015 through December 2016.

EMDR treatment was provided to the two groups, the intensive daily group receiving EMDR therapy twice each day for 10 days, and the weekly treatment group. The weekly therapy group consisted of veterans previously treated at veteran centers and local soldiers. Both groups were treated with the EMDR therapy standard protocol. The EMDR intensive successive days therapy model was established in 2005 designed to provide 50 min sessions twice a day for 5 days, with a minimum of 3 h between each session. Fifty-minute sessions were extended to 60 min as needed. Participants in the daily intensive group traveled from out of state. They frequently bore physical wounds from combat as well as psychological distress. Prior to being referred to Soldier Center for EMDR treatment, most of the participants in the daily intensive program had been treated, with varying degrees of success, by means of other models of psychotherapy. Early referrals came from Army Warrior Transitions Units (WTUs) with clients who had completed one and sometimes two combat tours of duty during Operation Iraqi Freedom (OIF) and Operation Enduring Freedom (OEF). As veterans experienced additional deployments the number of traumatic memories increased, producing greater symptom pathology. The daily treatment was expanded to 10 days to treat the expanding number of traumatic memories accompanied by increasing symptom severity. This allowed for the treatment of additional disturbing memories while the veteran was already in the area.

The daily intensive treatment allowed for the most distressing memories to be treated during the morning session with the afternoon session used to complete any residual material remaining from the earlier session while insuring the client finished the day with adequate stabilization and calmness. This approach provided for any residual traumatic material to be addressed within a few hours of each previous session.

Participants in the intensive daily treatment had been previously treated with other models of psychotherapy. Their personal motivation for treatment was assessed with respect to the impact of previous treatments on their expectations of current treatment outcomes.

Although most acknowledged being skeptical that additional treatments would work, all were willing to try another one since they did not want to live with their PTSD symptoms. Response to previous treatments reportedly ranged from compliance to veterans being so upset at the end of sessions they would “go out and get drunk,” which enhanced their alcohol dependency.

Other veterans reported prematurely dropping out of therapy to avoid the intensity of treatment. Some soldiers reported having completed treatment with other therapies ranging 2–4 years previously with disappointing results.

Treatment for both groups, successive days and weekly, presented a symptom-focused, time- limited approach with the goal of achieving the most effective results in the limited time available. Participants in the weekly treatment group occasionally missed an appointment due to work and pressing personal requirement. Persons traveling from out of state for the 10-day intensive program were expected to proceed with the process with little if any interruption since they were at their location specifically for treatment. No appointments were missed by the successive days intensive group. Both the weekly and daily intensive groups were treated with the standard eight-phase protocol of EMDR therapy. Lodging was provided in military barracks for junior-ranking enlisted soldiers participating in the intensive treatment condition.

Officers and non-commissioned officers (NCOs) were authorized by their military units to lodge at local hotels.

Veterans in the intensive daily group were referred because previous psychotherapy models had demonstrated limited success in treating them. While all of the participants in the intensive daily group had been previously treated with other models of psychotherapy prior admittance to Soldier Center, this was the case for only four of the members of the weekly treatment group.

The weekly group received 50–60 min sessions. In comparison, the successive days sessions during the 10-day intensive format lasted 75 min provided twice daily. The difference in session time between treatment groups was due to the intensive treatment group using an extended closing period to insure the client’s self-regulation and stabilization at the end of each intense session. Weekly group participants lived locally and presumably benefited from their already established support networks.

### Measures

All participants in the intensive daily treatment group had been screened and diagnosed with PTSD by mental health providers prior to their referral to Soldier Center. Four of the 15 participants in the weekly treatment had received a previous diagnosis of PTSD by referring agencies, along with prior treatment, before their referral to Soldier Center. Participants completed the evaluation process with an initial intake appointment. All met the diagnostic criteria for PTSD as outlined by the Diagnostic and Statistical Manual- IV (American Psychiatric Association [APA], 1994) and the Diagnostic and Statistical Manual-5 (American Psychiatric Association [APA], 2013), as reviewed by an independently licensed mental health practitioner who also provided the EMDR treatment. Each participant completed a packet of psychometric self-reports. The packet included the PTSD Checklist (PCL-M, now PCL-5), Impact of Events Scale-Revised (IES-R), Beck Depression Inventory (BDI), Beck Anxiety Inventory (BAI), and the Dissociative Experiences Scale II (DES-II). To meet the selection criteria for a PTSD diagnosis, measures for the PCL-M required a minimum score of 50 (Norris and Hamblen, 2004) and the PCL-5 a score of 33 (Department of Veterans Affairs [DVA], 2017). Additionally, the IES-R provided an independent measure a part from either the PCL-M or PCL-5. A cutoff score of 33 for the IES-R was used as a baseline for PTSD (Creamer et al., 2003; Beck et al., 2008).

Selection required all participants to have completed treatment and submitted a follow- up evaluation.

The PTSD Checklist (PCL-M) was used to validate the participants’ previous PTSD diagnosis made by their referring agencies (Norris and Hamblen, 2004). At the time of the treatment the use of the PCL was deemed more practical in a busy outpatient treatment center than the time-consuming CAPS (Foa and Tollin, 2006). The PCL (Weathers et al., 1993) is an easily administered self-report rating scale for assessing the 17 DSM-IV symptoms of PTSD. The measure has proven to maintain excellent test-retest reliability, with strong internal consistency. With a substantial correlation with other measures of PTSD, including the PK scale of the MMPI-2, the Mississippi Scale, and the IES, it provides an excellent tool. The PCL is known to provide good diagnostic utility when used as a continuous measure (Australian Centre for Post Traumatic Mental Health, 2016).

Change in the Diagnostic and Statistical Manual-IV (DSM-IV) to the Diagnostic and Statistical Manual-V (DSM-5), required a change in the PTSD Checklist with the introduction of the PCL-5. However, as the National Center for PTSD discovered, “PCL-5 scores are not compatible with PCL for DSM-IV scores and cannot be used interchangeably” (National Center for PTSD, 2016a,b, p. 1). Hoge et al. (2016) found that, based on responses to the new version, at least one-third of persons diagnosed with PTSD using the DSM-IV version do not meet criteria for PTSD according to the DSM-V definition. This change resulted in some participants in this study being diagnosed with a DSM-IV criteria and others using the DSM-V. Due to such disparity, the IES-R was relied on in this study for consistent measures across the cases.

During pre- and post-treatment, the Beck inventories (Beck et al., 2008) measured co-occurring levels of anxiety and depression. Depression characteristics and attitudes were measured with the Beck Depression Inventory. Anxiety, with components of cognitive and somatic functioning, was measured by the Beck Anxiety Inventory. Its cognitive subscale provided a measure of fearful thoughts and impaired cognitive functioning, and the somatic subscale measured the symptoms of physiological arousal. These inventories were included for further studies but not used in the current report. Previous follow-up studies at Soldier Center found that using the Beck Inventories as follow-up measures was problematic since both depression and anxiety inventories reflect the on-going life experiences in the client’s life at the time of the follow-up evaluation. Life experiences, including grief due to family deaths, divorces, and positive experiences such as births, and graduations, influence outcome measures.

Dissociative exhibitions, reflecting the participant’s ability to be mentally present and consciously focused, were measured by the Dissociative Experiences Scale (DES). Bernstein and Putnam (1986), authors of the scale reported that a score of 31 was associated with PTSD, whereas a score of 36 was the mean for dissociation-not otherwise specified.

### Assessment Points

All participants were assessed at three points during treatment at Soldier Center: pre- and post-treatment and 1-year follow-up. The initial intake occurred on arrival at Soldier Center and the second at the completion of the treatment plan. One year later a third assessment was conducted. Some treatment participants maintained occasional contact with the center following treatment while most assimilated into a routine at home with no contact with Soldier Center staff.

### Treatment Procedures

Previous published research indicate that EMDR therapy can resolve a single-incident trauma memory in three to-six sessions 77–100% of the time (Wilson et al., 1995, 1997; Marcus et al., 1997, 2004), while multiple combat trauma experiences generally require 12 or more sessions (Russell and Figley, 2013). All participants in both groups had been deployed multiple times, and demonstrated dissociative exhibitions ranging from loss of present awareness for a period to acting-out behavior while re-experiencing a traumatic memory. Moral injury issues were frequently presented as being attached to traumatic memories involving the violation of one’s personal values.

Both treatment formats incorporated the standard EMDR eight-phase approach (Shapiro, 2001, 2018). This approach includes: (1) History Taking; (2) Preparation; (3) Assessment; (4) Desensitization (reprocessing of the memory); (5) Installation; (6) Body Scan; (7) Closure; and (8) Reevaluation. The Adaptive Information Processing (AIP) model provides the theoretical underpinning of EMDR therapy. Pathology is viewed as the results of unprocessed, maladaptively stored memories. When memories remain unprocessed they continue to carry a highly emotional charge which, when triggered can create symptoms of PTSD and other disorders. EMDR therapy treats disturbing memories of the past, as well as present triggers, and future templates that prepares the person to effectively manage similar situations in the future. Effective treatment results in the diminishing of emotionally charged reactivity, the enhancement of positive self-referencing thoughts, and the clearing of somatically experienced body sensations associated with the targeted memory. Treatment plans consist of a list of disturbing memories associated with the client’s presenting symptoms. The list is developed during History Taking and self-regulation is developed during the Preparation phase. Finally, each disturbing memory listed in the treatment plan is treated in phases 3–8.

Many military active duty candidates for treatment in the intensive daily program were referred by a mental health manager at a military installation’s Warrior Transition Unit (WTU). Other participants were veterans discharged from active duty military who had been in treatment programs provided by the Department of Veterans Affairs and who learned of the EMDR therapy intensive treatment program. Initial inquiry was made by the case manager or sometimes by the veteran. Summaries of previous treatments were provided by the referring agency. The veteran was then given an assessment PTSD packet consisting of the PCL- M/PCL-5, IES-R, BAI, BDI, and DES-II. Participation was limited to persons who met the diagnostic criteria for PTSD. When active addiction was noted, the candidate was required to have been clean and sober for 6 months, with no active use at the time of the intensive treatment. While EMDR therapy protocols have been successively used in the treatment of addictions, the intensive daily treatment protocol does not allow time to treat both addictions and PTSD-related issues.

Admission into the intensive daily treatment program included a 50-min phone interview between the veteran client and the EMDR therapist. Discussion points included the veteran’s reason for seeking the intensive treatment, their motivation for treatment/change, any secondary gain issues, presenting symptoms, suicidality concerns, any dissociative features such as flashbacks, night terrors/nightmares, number of deployments, other challenging life events (beginning with childhood), relationship with parents and others, social activities and networks, who they can count on, any addiction history, moral injury issues, legal problems, significant losses, anniversary dates with mood changes, social and intra-personal stability, what they do to relax. Key points in each interview included the veteran’s motivation for change and ability to self-regulate when distressed. Persons unable to self-regulate were encouraged to do additional work with their local therapist to enhance self-regulation skills prior to coming for treatment.

Upon arrival at Soldier Center, veterans participating in the intensive daily program met with the designated EMDR therapist to begin the initial intake process. The first session was devoted to the History taking (Phase 1) with the client. This phase consists of a broad psychosocial interview in which the therapist learns what life has been like for the client. With a time-limited, symptom-focused approach, identifying the client’s goal(s) in seeking treatment as well as the presenting symptoms were noted. Introduction of the treatment plan addressed how treating specific disturbing memories can result in the resolution of the identified symptoms in accordance with the AIP theory underpinning EMDR therapy. The History- taking session culminated with two lists: a list of positive achievements in the person’s life, known as the “resource list,” and a list of disturbing life events which, when triggered, generated client disturbance. The list of distressing life events formed the basis from which the treatment plan was developed. It identified intrusive memories of past disturbing events that continued to interfere with the participant’s life. Additionally, current triggers and the apprehension of managing future events were included in the plan. The number of sessions required to treat each disturbing memory varied with each person, usually between one and three sessions per memory. The participant’s sense of security was continually addressed as effective treatment of PTSD requires the veteran to feel secure enough to address the vulnerability of recalling memories that are normally avoided.

Typically, in the second session of intensive daily treatment each veteran demonstrated the ability to relax or self-regulate by means of various relaxation techniques. The participant was introduced to the basic mechanics of EMDR treatment, which included bilateral stimulation (eye movement, auditory, or tactile stimulation), the introduction of a stop signal (should the client need to terminate the process during a session), and a relaxation exercise with a cue word.

With the completion of phases 1 (History taking) and 2 (Preparation), the veteran began treatment of the disturbing memories that were associated with the presenting symptoms. Each targeted disturbing memory in the treatment plan was treated with phases 3–8. For each veteran in the 10-day intensive daily treatment program, 6–10 disturbing memories were treated.

### Statistical Analysis

This study posed the questions: (1) does EMDR therapy administered twice a day have a positive effect on the veteran’s PTSD symptoms as measured by IES-R scores; (2) does EMDR therapy administered twice daily provide equivalent outcome results as EMDR therapy administered weekly for 18–20 sessions, as measured by the IES-R; and (3) does the treatment outcome last. **Table 1** demonstrates the significance in treatment outcome scores as measured by the IES-R. Descriptive statistics of scores collected at three time-points from each treatment group are reported. As can be seen in the table, the Intensive Daily group mean score at IES-R Pre-test was 53.2, which declined to a mean of 17.4 at the IES Post-test; the Follow-up IES score further reduced to 15.6. The standard deviations were 11.321 at IES Pre-test, 13.092 at IES Post-test and 6.967 at IES follow-up. The Weekly treatment group mean score at IES Pre-test was 51.8, which declined to a mean of 16.1 at the IES Post-test; the follow-up IES score increased to 17.7. The standard deviation was 8.326 at IES Pre-test, 6.923 at IES Post-test and 5.574 at IES follow-up. Variances between the two treatment groups were found to be approximately equal, with a significance level of 0.121. The mean scores between the groups were not significantly different, at 0.703 (**Table 2**).

**Table 1:** Descriptive statistics.

	Mean	N	Std. Deviation	Minimum	Maximum
Intensive daily (Pre)	53.20	15	11.321	29.00	66.00
Intensive daily (Post)	17.40	15	13.092	0.00	39.00
Intensive daily (FU)	15.60	15	6.967	4.00	30.00
Weekly (Pre)	51.80	15	8.326	35.00	63.00
Weekly (Post)	16.07	15	6.923	8.00	33.00
Weekly (FU)	17.73	15	5.574	5.00	25.00


**Table 2:** Independent samples *t*-test results for baseline measures of the two treatment groups.

	Levene’s test		*t*-test for equality of means						
		
	*F*	Sig.	*t*	*df*	Sig. (2-tailed)	Mean difference	Std. error difference	95% CI of the difference	
								
								Lower	Upper
Equal variances assumed	2.556	0.121	0.386	28	0.703	1.400	3.628	-6.032	8.832
Equal variances not assumed			0.386	25.716	0.703	1.400	3.628	-6.062	8.862


Two repeated measures ANOVAs were employed to compare the two independent groups (**Table 3**) with respect to pre-, post- and follow-up IES scores. For the Intensive Daily treatment group, the effectiveness of the treatment was demonstrated by the difference in IES scores among 3 time points, with statistically significant results (*F* = 105.21, *p* < 0.001). *Post hoc* evaluation of the group differences revealed a significant difference between Pre- and Post-test IES scores, while the Post-test IES and Follow-up IES scores were not significantly different (**Tables 3, 4**). This outcome was due to the improved scores measured at Post-test remaining only slightly changed at Follow-up.

**Table 3:** Tests of within-subjects effects for the successive day treatment group.

Source		Type III sum of squares	*df*	Mean square	*F*	Sig.	Partial Eta squared
IES successive day	Sphericity assumed	13493.200	2	6746.600	105.212	0.000	0.883
	Greenhouse-geisser	13493.200	1.604	8409.878	105.212	0.000	0.883
	Huynh-feldt	13493.200	1.780	7579.552	105.212	0.000	0.883
	Lower-bound	13493.200	1.000	13493.200	105.212	0.000	0.883
Error	Sphericity assumed	1795.467	28	64.124			
	Greenhouse-geisser	1795.467	22.462	79.933			
	Huynh-feldt	1795.467	24.923	72.041			
	Lower-bound	1795.467	14.000	128.248			


**Table 4:** Pairwise comparisons for the successive day treatment group.

(I) IES	(J) IES	Mean difference (I-J)	Std. Error	Sig.	95% CI for difference	
					
					Lower bound	Upper bound
Pre	Post	35.800^∗^	3.444	0.000	28.414	43.186
	FU	37.600^∗^	3.033	0.000	31.095	44.105
Post	Pre	-35.800^∗^	3.444	0.000	-43.186	-28.414
	FU	1.800	2.143	0.415	-2.796	6.396
FU	Pre	-37.600^∗^	3.033	0.000	-44.105	-31.095
	Post	-1.800	2.143	0.415	-6.396	2.796


*^∗^Indicates significant difference between pairs.*

The difference in the Weekly groups with regard to the IES-R scores among the 3-time points was found to be significant as well (*F* = 133.96, *p* < 0.0001). *Post hoc* evaluation of the group differences revealed a significant difference between pre- and post-test IES-R scores, while the post-test IES-R and Follow-up IES-R were not significantly different (**Tables 5, 6**).

**Table 5:** Tests of Within-Subjects Effects for the Weekly treatment group.

Source		Type III sum of squares	*df*	Mean square	*F*	Sig.	Partial eta squared
IES weekly	Sphericity assumed	12200.933	2	6100.467	133.964	0.000	0.905
	Greenhouse-geisser	12200.933	1.701	7174.429	133.964	0.000	0.905
	Huynh-feldt	12200.933	1.911	6383.200	133.964	0.000	0.905
	Lower-bound	12200.933	1.000	12200.933	133.964	0.000	0.905
Error	Sphericity assumed	1275.067	28	45.538			
	Greenhouse-geisser	1275.067	23.809	53.555			
	Huynh-feldt	1275.067	26.760	47.649			
	Lower-bound	1275.067	14.000	91.076			


**Table 6:** Pairwise comparisons for the weekly treatment group.

(I) IES	(J) IES	Mean difference (I-J)	Std. Error	Sig.	95% CI for difference	
					
					Lower bound	Upper bound
Pre	Post	35.733^∗^	2.566	0.000	30.229	41.237
	FU	34.067^∗^	2.824	0.000	28.009	40.124
Post	Pre	-35.733^∗^	2.566	0.000	-41.237	-30.229
	FU	-1.667	1.912	0.398	-5.767	2.433
FU	Pre	-34.067^∗^	2.824	0.000	-40.124	-28.009
	Post	1.667	1.912	0.398	-2.433	5.767


*^∗^Indicates significant difference between pairs.*

The results of the study revealed a significant decrease in IES-R scores from pre- to post-test for both the Intensive Daily and Weekly treatment groups. All participants fell below the cut-off score (33) for PTSD at post-test. These results correlated with the PCL-M/PCL-5 scores of the respective veterans. Additionally, the non-significant difference between post-test and follow-up IES scores indicates that the treatments were also effective in the long-term because the participants were able to retain their treatment gains.

To examine whether one treatment had a greater impact on the participants than the other, F statistics and their effect sizes were also compared. In this study, the following formula was used to calculate effect size for the repeated measures ANOVA:

$$\eta^{2}\;=\;{\mathrm{SS}}_{w}/\mathrm{SS}$$

where SSw is the sum of squares for the within-subject effect, and SSe is the sum of squares of the total error variance.

The effect size for the Successive Day treatment group is 13493.2/18366.8 = 0.735, and the effect size for the Weekly treatment group is 12200.9/14277.2 = 0.855. Both effect sizes were large. However, the Weekly treatment group evidenced a larger *F*-value as well as effect size, and therefore in the current study, the findings suggest that the Weekly treatment had a greater impact on participants with PTSD, while the Intensive Daily treatment demonstrated a slightly greater effect within a limited period of time, an effect that was maintained at 1-year follow- up.

## Results

This three-point assessment of two EMDR treatment groups suggest EMDR therapy, when administered twice daily during a 10-day period, produced significant improvement in symptom reduction as measured by the IES-R. The same conclusion was supported when evaluating weekly treatment with 18–20 sessions over a 5–6-month period. Both the intensive daily group and weekly treatment group have significant outcome results at post-test, with results being maintained at 1-year follow-up.

These findings indicate that EMDR intensive daily treatment may offer an accelerated treatment model for other therapy programs. As previously noted, all members of the intensive daily group had been treated for PTSD prior to their referral to Soldier Center. Three members of the intensive daily group had previously been treated with EMDR therapy unsuccessfully. Veterans with previous unsuccessful treatment experiences improved significantly in this treatment program. Effective treatment occurred with a change of therapists and treatment centers. The explanation for this observation is unclear. One the one hand, therapists in the current study demonstrated satisfactory treatment fidelity through the monitoring of their videotaped sessions. A meta-analysis of EMDR therapy research (Maxfield and Hyer, 2002) reported a significant positive correlation between effect size and treatment fidelity. However, there were no fidelity checks of the previous clinicians.

This study is limited by its sample size, with only two groups of 15 members in each condition evaluated. Future RCT research is needed with a larger sample size. However, the study does evaluate a select group of veterans whose multiple traumas, complex PTSD, and moral injury issues required 18–20 sessions for treatment. This exceeds the number of sessions normally required for the successful treatment of single-incident trauma and adverse life experiences. Future research should more rigorously examine this population.

An evaluation of each treatment format found that the Intensive Daily treatment and Weekly treatment conditions were equally effective, with a substantial decrease in symptoms and all of their participants losing their PTSD diagnosis. The intensive daily treatment format provided a rapid resolution of trauma with persons stable enough to self-regulate, while addressing their disturbing memories during a 10-day period. Similar results were found with participants in the weekly treatment group. The intensive daily condition was able to accommodate veterans suffering from extended histories of trauma such as persons serving in critical medical care positions who experienced up to 14 years of military and medical trauma during OIF/OEF assignments. In such cases the 10-day period was not sufficient to resolve all of their traumatic memories, but their major trauma memories were treated as one component of an expanded treatment plan designed to achieve an accelerated healing process of the primary trauma memories before returning home to continue weekly treatment with their local EMDR therapist. However, in all cases they no longer retained the diagnosis of PTSD after the 10 days of treatment. Further, no adverse experiences were precipitated by treatment in either condition. There was a 0% dropout rate in the intensive treatment group and 8% in the weekly group (due to relocation). These findings are consistent with those of a meta-analysis (Swift and Greenberg, 2014) reporting that EMDR therapy had a lower dropout rate than any other trauma-focused treatment that was evaluated.

It should be noted that all persons in this study completed their treatment plan in 18–20 sessions. Therapists operated from the principle: don’t open psychological wounds you do not have adequate time to resolve. In the beginning of Soldier Center’s intensive treatment program, the goal was to restore the veteran to a level of psychological functioning experienced prior to military service. It was common for a veteran to identify up to 10 combat traumas and one early life adverse event. If there were additional combat deployments with numerous traumatic memories, the goal was to identify and address those targeted disturbing memories that would provide the most psychological healing within the limited period of treatment time. For some veterans this meant that all their traumatic memories could be treated in the designated period. Other veterans with multiple combat deployments identified those disturbing memories that could be treated within the intensive period and then returned to their home region where follow-up treatment was provided by a local EMDR therapist on a weekly basis.

## Discussion and Conclusion

The research questions addressed in this study were: (1) does EMDR therapy administered twice daily ameliorate veterans’ PTSD symptoms; (2) does EMDR therapy administered twice daily provide equivalent outcome results as EMDR therapy administered weekly for 18–20 sessions; and, (3) does the treatment outcome persist? When comparing the pretreatment, post-treatment, and follow-up IES-R scores, two repeated measures ANOVA noted in the intensive daily group the difference on the three-points were significant (*F* = 105.21, *p* < 0.001). *Post hoc* evaluation of the group differences revealed a significant difference between pre- and post-test IES-R scores. The difference in scores between post-test and follow-up scores were not significant indicating the significant improvement if PTSD symptoms as measured by the IES-R at the end of the 10-day treatment. The twice daily treatment was found to be significant in ameliorating veterans’ PTSD symptoms.

EMDR therapy provided approximately equal outcome results when reviewing treatment results of daily and weekly treatment. At post-test the intensive daily treatment mean changed from 53.20 to 17.40 at post-test and 15.60 at follow-up indicating the treatment change at post-test was maintained at the 1-year evaluation. The weekly treatment changed from the IES-R mean of 51.80 at pre-test to 16.07 at post-test and 17.73 at 1 year follow-up. With a variance of 1.027 between the groups at pre-test, the between group variance at post-test was 1.330. Follow-up 1 year later noted a variance of 2.130.

Thte third question, does the treatment persist, was demonstrated at 1 year follow-up. Intensive daily treatment group improved from an IES-R mean of 17.40 at post-test to a mean score of 15.60 at 1 year follow-up. The weekly treatment group IES-R mean increased by 1.66 between post-treatment and 1 year follow-up. It can be stated, based on these findings that both treatments maintained their outcome significance at 1 year follow-up.

The intensive daily format and the weekly treatment approach both offer benefits for participating veterans. The intensive program can be formatted for inpatient treatment in VA medical centers. Likewise, active duty personnel can be temporarily transferred to facilities with seasoned EMDR clinicians who can complete treatment within two weeks. The intensive treatment allows any reactivity that might surface to be immediately addressed. Additionally, the intensive program provides momentum in the treatment. Little time is needed for getting caught up with events between sessions since only a few hours elapse between sessions. The time saved due to this momentum can be applied to further treatment work.

For some participants the weekly EMDR treatment provides a better fit for their time. They have difficulty getting away for a two-week period due to work or family responsibilities and finances. Weekly appointments allow for the emotional support of family and friends between sessions. The weekly treatment format provides time for psychological triggers to be identified between sessions and added to the treatment plan. This format also allows more time for trust and rapport to develop in the clinical relationship.

The intensive daily10-day treatment program offers many possibilities for treatment centers. However, the likelihood of obtaining the same outcomes in other treatment settings is yet to be determined. Based on the findings of this study, additional research using EMDR therapy in an intensive successive-days process is warranted.

An additional application of EMDR therapy in military personnel that merits further investigation is the treatment of phantom limb pain. An RCT (Rostaminejad et al., 2017) and multiple case studies (e.g., Russell, 2008; de Roos et al., 2010) have reported the elimination of phantom limb pain subsequent to EMDR treatment. As previously mentioned, the AIP model posits that the basis of PTSD and other disorders is the inappropriate storage of unprocessed memories (Shapiro, 2001, 2018). These memories retain the emotions, physical sensations, and beliefs that occurred at the time of the event. Phantom limb pain can resolve when the memory of the initial injury is targeted. Given the number of veterans suffering from this condition, additional RCTs on this pathology are recommended.

## Author Contributions

The author confirms being the sole contributor of this work and approved it for publication.

## Conflict of Interest Statement

The author declares that the research was conducted in the absence of any commercial or financial relationships that could be construed as a potential conflict of interest.
