# Supplementary material for: Effective Treatment of Veterans With PTSD: Comparison Between Intensive Daily and Weekly EMDR Approaches
Source: Front Psychol. 2018 Aug 24;9:1458. doi: 10.3389/fpsyg.2018.01458 (PMC6117416; doi:10.3389/fpsyg.2018.01458)
Supplement: Supplementary file 1 [file Presentation_1.pdf]

Effective treatment of veterans with PTSD: Comparison between intensive daily and weekly EMDR approaches

Supplement outlining the clinical protocol

E. C. Hurley, DMin, PhD

The effectiveness of EMDR therapy in treating veterans diagnosed with PTSD was evaluated in this study using two treatment formats: (1) intensive daily EMDR treatment twice a day during a 10-day period and (2) weekly treatment sessions. The study used archived outcome data previously collected and stored at Soldier Center. Both formats provided 18-20 treatment sessions of EMDR therapy to veterans diagnosed with PTSD.

Questions addressed in the study included: (1) does EMDR therapy administered twice daily ameliorate veterans' PTSD symptoms; (2) does EMDR therapy administered twice daily provide equivalent outcome results as EMDR therapy administered weekly for 18-20 sessions; and (3) does the treatment outcome persist at one year?

Clinical protocol

1. Referral
   Veterans included in this program were referred for treatment from military Warrior Transition Units, active duty military mental health case managers, and, on some occasions by family members who learned of the treatment programs at Soldier Center, Clarksville, TN.

   Soldier Center treats three (3) categories of PTSD diagnosed veterans: Category 1 is acute stress, adverse life experiences, and simple PTSD. This category is normally treated within 6-12 sessions of EMDR therapy; (2) category 2 consists of veterans with symptoms more commonly associated with C-PTSD and moral injury requiring 12-20 sessions for complete resolution of their symptoms; and (3) category 3 consists of veterans requiring 20+ sessions of EMDR therapy. Category 3 includes clients with severe PTSD symptoms who have chronic histories of exposure to severe traumatic events such as medical personnel who have spent years in medical assignments in combat zones working in emergency medicine and combat soldiers who have multiple deployments to war zones. Clients included in this study consisted of category 2 veterans.

2. Intake
   Veterans from out of state were considered for the intensive, daily treatment program. Referred persons were evaluated before being accepted as a candidate in the daily program. The PTSD Checklist and Impact of Events Scale-Revised (IES-R) and Dissociative Experiences Scale-II (DES-II) were administered by the referring agency. The

collected data was sent electronically to Soldier Center for review to insure the potential candidate met diagnostic criteria for PTSD. A 45-minute phone interview was conducted to insure the client was appropriately motivated for treatment, had the ability to emotionally self-regulate, rule out secondary gain issues which might impede treatment. Presenting issues/symptoms were discussed and a brief history focusing on military deployments, family of origin attachment history and social/emotional support system. Disorders such as alcohol/drug dependency, depression and suicidality/homicidality were ruled out.

On arrival at Soldier Center, Clarksville, TN, the client completed the PTSD packet consisting of the PCL-M/PCL-5, Impact of Events Scale-Revised, Beck Anxiety Inventory (BAI), Beck Depression Inventory-II (BDI-II), and the Dissociative Experiences Scale (DES-II).

Veterans living in the regional area near Soldier Center were primarily referred by military mental health managers, veterans' programs, and self-referred. These clients received EMDR therapy on a weekly basis. They completed the same PTSD packet as described above during their initial intake session.

All participants were briefed insuring the clients' confidentiality would be safe guarded. Consent to use the collected data to improve therapy for veterans in future treatment was sought. Verbal consent for using the collected in future research publications was sought and gained with the understanding all data would be anonymized at the source.

3. Presentation of treatment plan during first or second session.
   The client's presenting problem(s)/issues were identified in the first session, clarifying what the veteran wants to change as a result of treatment. Using the EMDR theoretical model, the Adaptive Information Processing model, the therapist completed history taking (1-3 sessions) particularly looking for distressing events in the client's life which contribute to the presenting problem(s). A list of positive resources were identified, i.e., memories of achievements/successes the client has accomplished which, when recalled, help the client to feel positive about themself. A list of presenting symptoms and past events they relate to is developed. It was explained that the therapist is looking for memories of past events which, when reminded, trigger the veteran to react. It was further clarified as "things that have happened in the past, which when reminded, they still bother you."

4. From the above list those disturbing memories (and the current triggers) are connected and identified for treatment with EMDR therapy. Those unresolved memories of past events and present triggers connected with the presenting problem are identified, developed as a treatment plan and treated with EMDR therapy. EMDR therapy is an

eight-phase approach. The history taking (phase 1) and treatment plan are collaboratively developed with the veteran in one to three sessions.

5. Once the treatment plan is developed, EMDR therapy's preparation (phase 2) provides the client with an introduction to EMDR therapy by "setting up the mechanics of treatment, i.e., introducing the concepts of bilateral stimulation with three different modalities (auditory, tactile, and eye movement). A stop signal is established in case the client needs to stop the process. Informed consent is provided, and the client is introduced to self-regulation relaxation techniques to insure he/she can relax and self-regulate.

6. With the client demonstrating their ability to self-regulate, EMDR therapy is used to treat each of the disturbing memories and present trigger incidents identified in the treatment plan (see #4 above). EMDR therapy is a 3-prong approach which treats disturbing memories of past events, present triggers, and the use of the future template preparing the client to effectively manage their life in future situations.

7. EMDR therapy is used to treat each target memory identified in the treatment plan using EMDR phases 3-8. Those phases are:

   Phase 3: Assessment – accessing the disturbing memory and activating it for treatment by asking 7 questions.

   Phase 4: Desensitization – this is the reprocessing of a disturbing memory using bilateral stimulation. The goal of this phase is to reduce the subjective level of disturbance (SUD) to 0 on a scale of 0-10. This phase can take 1-4 sessions as each client processes differently.

   Phase 5: Installation (of the positive cognition). A scale of 1-7 is used to insure how valid the positive self-referencing belief (Validity of Cognition or VOC) develops, making it as strong as possible.

   Phase 6: Body Scan. This phase checks for somatically stored aspects of the trauma memory which is represented by physical sensations experienced in the body. Use of bilateral stimulation is used to dissipate or clear these sensations. Having a clear body scan is a necessary part of the treatment.

   Phase 7: Closing – there are two types of closing, incomplete (when you still have additional work to treat the targeted memory) and complete session (when you have a SUD of 0, and VOC of 7, and a clear body scan.

   Phase 8: Reevaluation – beginning the next session by evaluating where the client is presently in regard to the treatment work from the previous session.

8. Each memory listed in the treatment plan (see #2 and #3 above) is treated phases 3-8 resulting in the client having a subjective unit of disturbance (SUD) of 0, a Validity of Cognition (VOC) of 7, and a clear body scan.

9. At final termination (session 18-20 in this study) a posttest assessment of taken using the PTSD Checklist, the IES-R, and the DES-II. These scores were entered in the Excel PTSD spreadsheet.

10. For follow-up at 12 months the IES-R was sent to clients for their follow-up evaluation. Scores were entered in the Excel PTSD spreadsheet and archived. Name, age, dates of treatment, number of sessions was entered for archiving.

11. Archived records were retrieved and evaluated. Collected data was used in the two research groups, daily successive days treatment and the weekly treatment formats. Since a total of 15 veterans had been treated in the daily treatment program and equal number of clients treated weekly was accessed as well. The first 15 participants in the weekly treatment format were selected for the weekly treatment group.

12. A t test and repeated measures ANOVA were used to evaluate the archived research data. Findings supported the efficacy of EMDR therapy in an intensive daily treatment format providing EMDR therapy twice a day over a 10-day period. Treatment results were maintained at one-year follow-up.
